# Effect of Ultrafine Cement (UFC) on the Corrosion Resistance of Cement Soil in Peat Soil Environment

**DOI:** 10.3390/ma16165520

**Published:** 2023-08-08

**Authors:** Yongfa Guo, Jing Cao, Huafeng Sun, Wenyun Ding, Guofeng Hua, Wei Wei, Siyang Huang

**Affiliations:** 1 Kunming Survey, Design and Research Institute Co., Ltd. of CREEC, Kunming 650200, China; guoyf@ey.crec.cn (Y.G.); dingwy@ey.crec.cn (W.D.); 2 Faculty of Civil Engineering and Mechanics, Kunming University of Science and Technology, Kunming 650500, China; sunhuafeng@stu.kust.edu.cn; 3 Sichuan Xinguangjian New Materials Technology Co., Ltd., Chengdu 610000, China; scxgj002@163.com; 4 Guangxi Zhuang Autonomous Region Testing Institute of Product Quality, Nanning 530000, China; aihuaww702604@126.com

**Keywords:** peat soil, ultrafine cement (UFC), cement soil, durability, strength test, micro test

## Abstract

Many peat soils are distributed around plateau lakes, and the reinforcement of peat soils with high organic matter content by ordinary cement cannot meet the actual engineering requirements. In order to obtain better mechanical properties and durability of the reinforcement, this experiment prepared peat soil by mixing humic acid reagent into the alluvial clay soil with low organic matter content. The cement soil samples were prepared by adding cement and ultrafine cement (UFC) by stirring method; the samples were then soaked in fulvic acid solution to simulate the cement soil in the peat soil environment. Using the unconfined compressive strength (UCS) test, scanning electron microscope (SEM) test, and pores and cracks analysis system (PCAS) test, the effect of UFC content change on cement soil’s humic acid erosion resistance was explored, and the optimal UFC content range was sought. The results of the UCS test show that with an increase in immersion time, the strength curves of cement soil samples gradually increase to the peak strength and then decrease. Significant differences in the time correspond to the peak strength, and the overall presentation is two processes: the strength enhancement stage and the corrosion stage of the sample. The incorporation of UFC makes the cement soil in the peat soil environment exhibit excellent corrosion resistance, and the optimal UFC content is 10%. The results of the SEM and PCAS tests show that the microstructure of cement soil after immersion time exceeds 90 days, increases with an increase in immersion time, and its structural connectivity gradually weakens. The excellent characteristics of UFC particles, such as small particle size, narrow particle size distribution, fast hydration reaction rate, high hydration degree, and many hydration products, weakened the adverse effects of humic acid on the cement soil structure to a certain extent. Therefore, although the number of macropores increases, they are not connected. It still presents a relatively compact honeycomb overall structure, which correlates well with the UCS results.

## 1. Introduction

The area around the Dianchi Lake and Erhai Lake in Kunming is an ancient lake and swamp area with deep Quaternary deposits. Its particular geographical location and plateau climate make the peat soil widely distributed in this area [1]. Peat soil contains a large amount of humic acid. For traditional cement-based materials that have been in a saturated and acidic environment for a long time, the hydration process is slow, the hydration products are dispersed, and the reinforcement strength is rapidly attenuated by humic acid erosion. Traditional cement soil reinforcements inevitably suffer from reduced mechanical properties and poor durability. Therefore, seeking a green and low-carbon treatment method to enhance cement soil’s corrosion resistance and improve cement soil’s durability has become a matter of great concern to academic circles and the industry.

The smaller the cement particle size and the larger the specific surface area, the faster the hydration rate of cement and the faster the strength will be exerted [2]. Reducing the particle size of cement particles and optimizing the type and dosage of composite curing agents is an effective method to improve cement soil’s working performance. Foteini Kontoleontos et al. [3] studied the influence of cement fineness on the physical and chemical properties and microstructure of cement. The results showed that the impact of cement fineness on compressive strength was mainly manifested in the early stage, and the ultra-fine cement showed a relatively dense form from the beginning of curing—microstructure. Wang Shuren [4] used ultrafine Portland cement (UPC) to reinforce sludge. The results showed that UPC-modified sludge could produce more hydration products, higher compressive strength, remarkable early strength characteristics, and stronger deformation resistance under the same conditions. Compared with ordinary cement (OPC) modified sludge, the strength of UPC-modified sludge after curing for one day was about four times greater than that of ordinary Portland cement (OPC) modified sludge. Research by Juan Carlos Arteaga-Arcos et al. [5] showed that adding ultra-fine cement can optimize the particle size distribution of cement, resulting in better particle packing density and lower porosity. Its replacement rate for ordinary cement is 30% to 40%, and the improvement of mortar strength is the most significant. Yi Shi et al. [6] described the influence of cement particle fineness on cement hydration. They concluded that cement fineness significantly impacts the early hydration process. The smaller the cement particle size, the higher the early compressive strength. The compressive strength of ultra-fine cement mortar with a particle size of 6.8 μm can reach 55.94 MPa after curing for 24 h, 118% higher than that of ordinary cement mortar. IB Celik [7] studied the effect of particle size distribution, uniformity, and specific surface area of cement particles on cement’s performance and showed that cement’s fineness would affect the early stage of hydration reaction and early strength development. Zheng Xiyao [8] studied the effect of ultra-fine cement on the early mechanical properties of solidified soft soil. The results showed that with a decrease in cement fineness, the hydration reaction rate of composite curing agents increased, and the number of hydration gel products increased. The structure becomes compact, and the cured soft soil’s unconfined compressive strength and elastic modulus increase. The test results of Chen Xudong [9], who discussed the influence of particle size distribution on cement hydration kinetics, showed that cement’s fineness significantly influenced hydration within seven days. The non-evaporating water content of ultra-fine cement paste was higher than that of ordinary cement paste. The higher the cement paste, the higher the hydration degree. During the seven test ages, the superfine cement produced a higher gel/space ratio, and its porosity was lower than that of ordinary cement paste.

In summary, scholars have conducted much research on UFC modification. However, research on the influence of UFC on the mechanical properties of cement soil in a peat soil environment is rare, and the long-term durability of UFC in a peat soil environment is even less so. Therefore, in this study, peat soil was made by adding humic acid to the alluvial and diluvial clay soil with low organic matter content and then mixed with cement and ultrafine cement (UFC) by stirring method to make cement soil samples and placed in fulvic acid solution to simulate the actual working environment of cement soil. The UCS, SEM, and PCAS micro-semi-quantitative tests were conducted on cement soil. The study will initially reveal the influence of UFC on the corrosion resistance of cemented soil in the peat soil environment and put forward an optimal UFC content that is economical and can effectively improve the corrosion resistance of cemented soil to humic acid, providing theoretical guidance for practical engineering.

## 2. Test Design

### 2.1. Experiment Material

**Figure 1:**
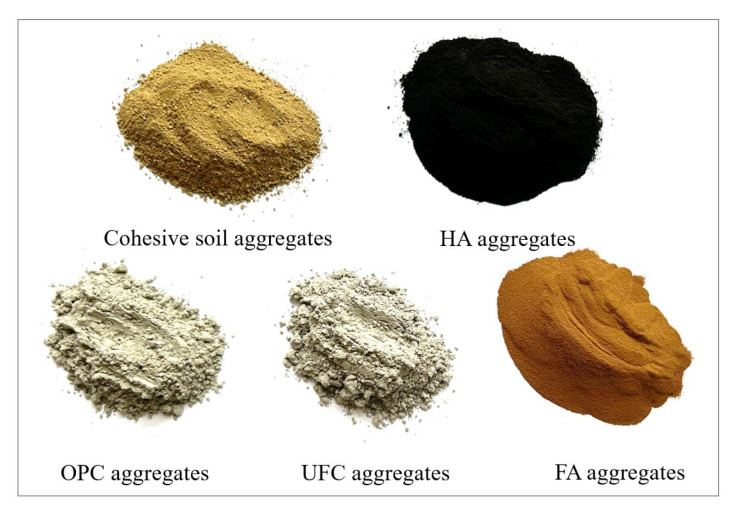
Test material.

The appearance of soil samples, humic acid (HA) reagent, fulvic acid (FA) reagent, Ordinary Portland Cement (OPC), and ultrafine cement (UFC) used in the test are shown in Figure 1.

**Figure 2:**
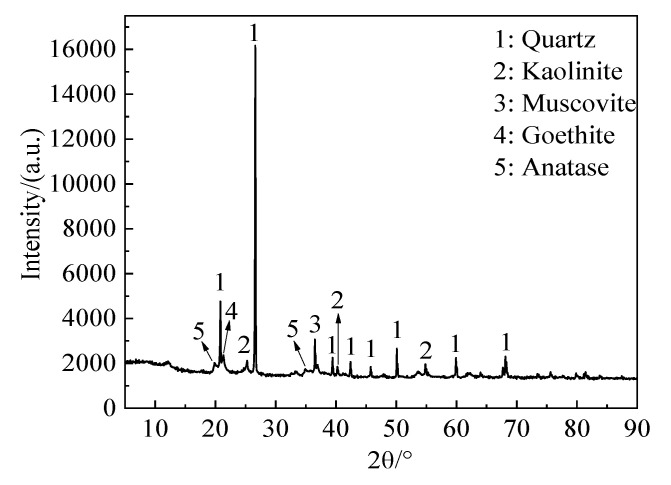
X-ray Diffraction Pattern of Cohesive soil.

**Figure 3:**
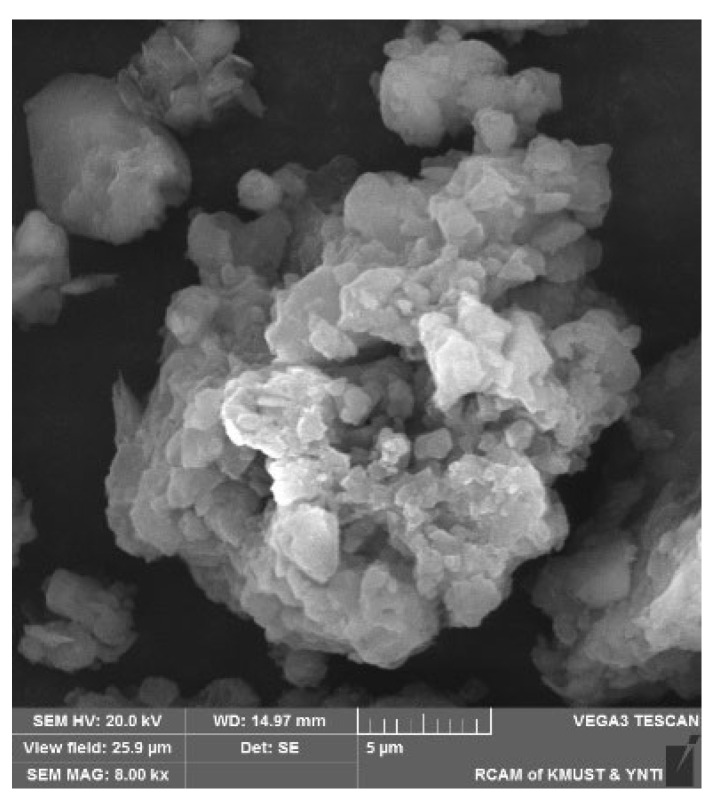
Microstructural image of humic acid aggregates [10].

**Figure 4:**
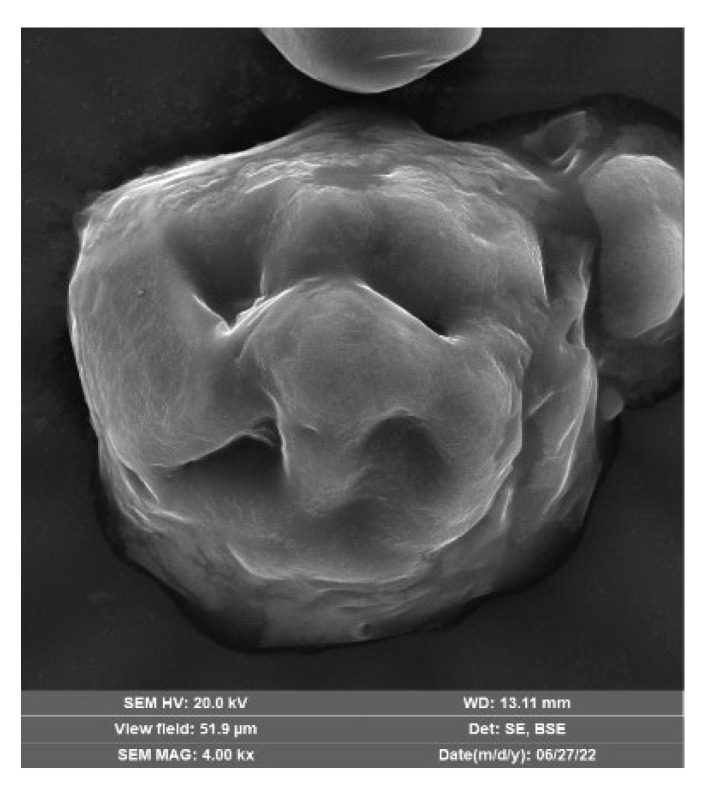
Microstructural image of fulvic acid aggregates.

**Figure 5:**
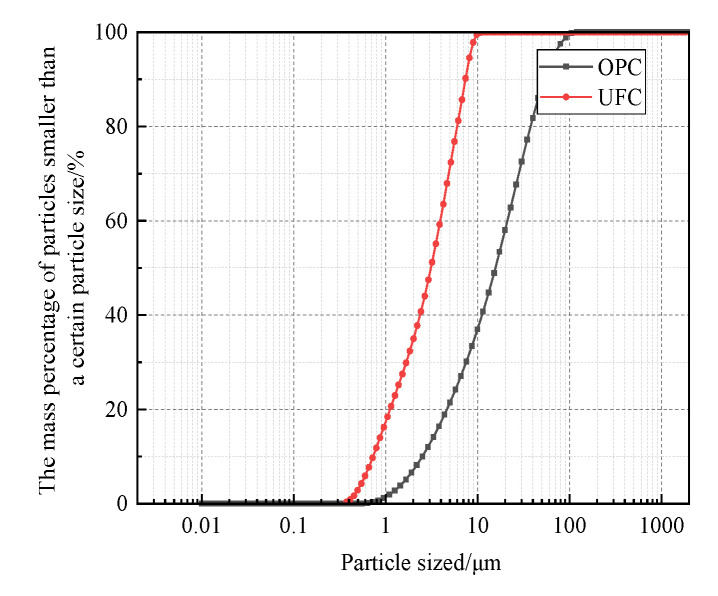
Cumulative distribution curves of OPC and UFC particle size gradation.

**Figure 6:**
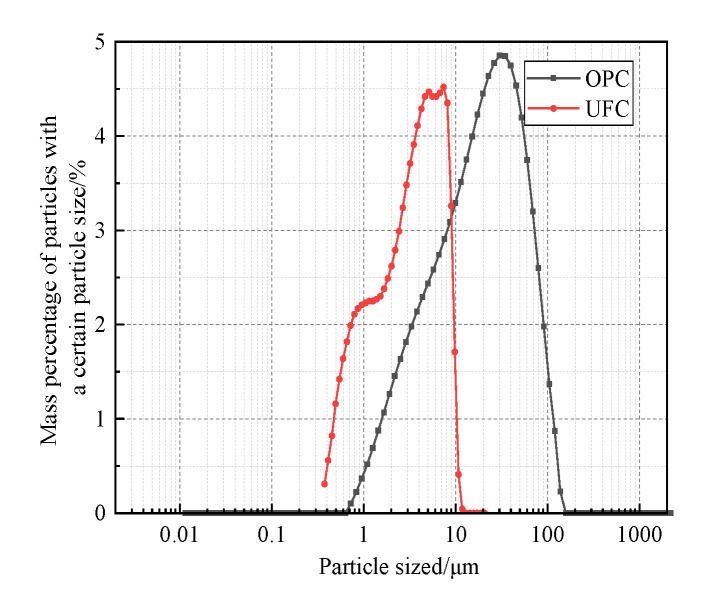
OPC and UFC particle size distribution curves.

The basic information of the test materials is as follows:

(1) The soil used for the test is the alluvial and diluvial clay soil on the north slope of the Jingyuan Student Apartment, Chenggong Campus, Kunming University of Science and Technology. The undisturbed soil sample is brownish-yellow. The basic physical property indicators are shown in Table 1. The base parameters of the soils in this table will be used as input conditions for sampling in subsequent tests. The X-ray fluorescence (XRF) analysis results are shown in Table 2. The X-ray diffraction test (XRD) results are shown in Figure 2, which shows that the main crystal phases of alluvial clay soil are quartz, kaolinite, muscovite, goethite, and anatase. The test results show that the soil sample has a single composition and low organic matter content. Therefore, selecting this soil sample as a raw material has little influence on the test results.

(2) Tianjin Guangfu Chemical Reagent Factory (Tianjin, China) produces the humic acid (HA) reagent, and the actual humic acid content is 41.68%. Its microstructure is shown in Figure 3, it can be seen that the humic acid aggregates are loose and porous, and its structural connection is weak.

(3) Pingxiang Hongtudi Humic Acid Co., Ltd. (Pingxiang, China) produces the fulvic acid reagent. The actual content of fulvic acid is 60%. Its microstructure is shown in Figure 4. It can be seen that the fulvic acid aggregates are colloidal.
(4) Distilled water is used for mixing samples and fulvic acid and soaking solution in the test.
(5) The cement selects Shilin brand P·O 42.5 grade ordinary Portland cement (OPC) is produced by Huaxin Cement Co., Ltd. (Wuhan, China).
(6) Ultra-fine cement (UFC) is made as the abovementioned ordinary cement through physical processing and grinding, and its specific surface area is more significant than 9000 cm^2^/g and d90 < 10 μm. The main chemical components and mass fractions of the two types of cement are shown in Table 3, the cumulative distribution curve in Figure 5, and the particle size distribution curve in Figure 6. Through data analysis, it can be seen that the main chemical components and contents of the two are the same. However, UFC has a more concentrated particle size distribution than OPC.

### 2.2. Test Method and Sample Preparation

According to research, the content of organic matter in peat soils around Dianchi Lake and Erhai Lake in Yunnan ranges from 10.73% to 75.09%, the total amount of humic acid ranges from 7.15% to 50.06%, and the content of humic acid ranges from 2.36% to 28.13% [11]. Therefore, the humic acid content in this test was 0%, 15%, and 30%, respectively, and the cement mixing ratio was 20%. Moreover, the cement soil sample was soaked in fulvic acid solution (continuously adding fulvic acid to keep the pH value of the fulvic acid solution constant) and distilled water. The soaking time of the sample is set to 28 d, 90 d, 180 d, 270 d, and 365 d. In order to meet the sampling conditions and the essential characteristics of peat soil, the water content ω = 24% is determined and controlled, void ratio e = 1.2. The specific experimental design is shown in Table 4.

The test was based on the “Standards for Geotechnical Test Methods” (GB/T50123-2019) [12]. The amount of raw materials was calculated according to Formulas (1) to (4). Cement soil samples were prepared using a three-part mold with an inner diameter of d = 39.1 mm and a height of 122 h = 80.0 mm. Then, the sample was placed in a curing box with a humidity of 95 ± 2% and a temperature of 20 ± 2 °C for ten days. After curing, the samples were soaked in a fulvic acid solution.

(1) $$\lambda =\frac{m_{\left(HA\right)}}{m_{\left(soil\right)}+m_{\left(HA\right)}}\times 100\%$$

(2) $$\beta =\frac{m_{\left(OPC\right)}+m_{\left(UFC\right)}}{m_{\left(soil\right)}+m_{\left(HA\right)}}\times 100\%$$

(3) $$\gamma =\frac{m_{\left(UFC\right)}}{m_{\left(OPC\right)}+m_{\left(UFC\right)}}\times 100\%$$

(4) $$m_{\left(water\right)}=(m_{\left(soil\right)}+m_{\left(HA\right)})\times \omega$$

In the formula: *λ*—the amount of humic acid added, %; *m*_(*HA*)_—the mass of HA reagent particles, g; *m*_(*soil*)_—the mass of soil particles, g; *β*—the cement mixing ratio, %; *m*_(*OPC*)_—the mass of OPC, g; *γ*—the UFC mixing ratio, %; *m*_(*UFC*)_—the mass of UFC, g; *m*_(*water*)_—the mass of distilled water in the sample, g; *ω*—the moisture content of the sample, %.

### 2.3. Testing Process

In this study, the samples that have reached the soaking time are taken out, and the samples are pretreated to ensure they meet the test requirements. The samples were subjected to the UCS, SEM, and PCAS micro-quantitative tests. The specific test process is as follows:

(1) UCS test

The UCS test was performed with the help of the YSH-2 electric lime soil unconfined compression tester produced by Nanjing Ningxi Soil Instrument Co., Ltd. (Nanjing, China) The UCS is measured for the samples whose immersion time is 28 d, 90 d, 180 d, 270 d, and 365 d. The arithmetic means the value of the tested samples is taken as the UCS value of the group of samples. The axial compression rate of the test control instrument is 1.0 mm/min.

(2) SEM test

SEM test sample preparation and test process: first, the test group samples are taken out and placed in an oven to dry; a sample with a length, width, and height of 30 mm × 20 mm × 15 mm is cut out at the exact position of the sample, and the Czech TESCAN-VEGA3 full-body is used. An automatic scanning electron microscope is used to observe the microstructure inside the sample.

(3) PCAS microscopic quantitative test

The PCAS test is to preprocess the scanning electron microscope test photos. Using the microstructure processing program Pores and Cracks Analysis System (PCAS software Version 2.324) developed by the Department of Earth Sciences of Nanjing University, the corresponding microstructure parameter information was extracted, and the pore distribution law of the cement soil sample was obtained statistically.

## 3. Test Results and Analysis

### 3.1. Effect and Mechanism Analysis of UFC on the Corrosion Resistance of Cement Soil in the Peat Soil Environment

**Figure 7:**
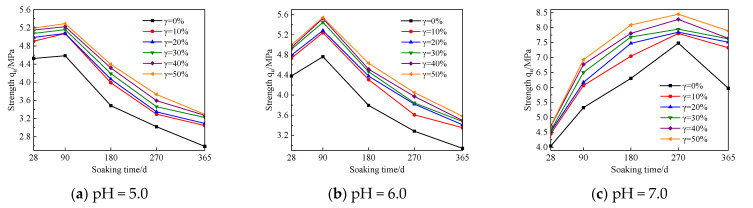
The relationship curve between the strength of the cement soil sample and soaking time (λ = 0%).

**Figure 8:**
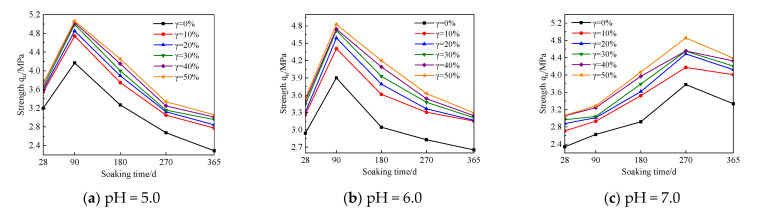
The relationship curve between the strength of the cement soil sample and soaking time (λ =15%).

**Figure 9:**
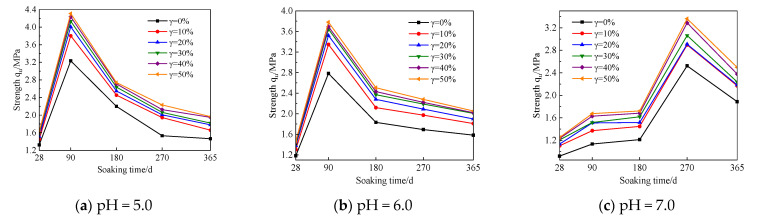
The relationship curve between the strength of the cement soil sample and soaking time (λ = 30%).

In order to explore the influence trend of UFC content changes on the strength of cement soil samples under different humic acid environments, this study regards the cement soil samples with the same UFC content as the same group of samples for comparative analysis. The relationship curves between sample strength and immersion time are shown in Figure 7, Figure 8 and Figure 9. The test results show: (1) With an increase in immersion time, the strength curves of the cement soil samples under different pH values of fulvic acid immersion solutions gradually increased to the peak strength and then decreased. That is to say, the strength change curve presents two stages, the stage of strength enhancement and the stage of corrosion. However, the immersion time corresponding to the peak strength significantly differs. The strength of the sample in the fulvic acid soaking solution reached its peak when the soaking time was 90 d, and its strength gradually decreased after the soaking time exceeded 90 d; while the strength of the sample in distilled water reached the peak when the soaking time was 270 d, and after the soaking time exceeded 270 d, its strength gradually decreases. (2) In the case of the exact pH of the saturated solution and the same amount of humic acid reagent added, as the amount of UFC increased, the strength curve increased significantly compared with the sample without UFC, and the strength curve increased significantly with the immersion time. From the perspective of long-term trends, it has specific corrosion resistance.

The combined action of UFC and fulvic acid causes a difference in the peak strength of the sample. The molecular configuration of fulvic acid is easily affected by pH value and ionic strength. The lower the pH value, the higher the ionic strength, which is more conducive to forming rigid spherical colloids of fulvic acid [10]. Research shows that [7,13,14] cement particle fineness can be divided into four intervals: less than 3 μm, 3–30 μm, 30–60 μm and greater than 60 μm. The fineness of cement particles has different effects on cement soil strength under soaking time. Cement particles smaller than 3 μm have a fast hydration rate and contribute significantly to cement soil’s early strength (1–3 d). The hydration of cement particles with a particle size of 3–30 μm mainly affects the strength of cement soil from 28 days to 90 days; the coarse cement particles with a particle size of 30–60 μm are slightly hydrated at 28 days, which mainly affects the strength after 90 days. Cement particles larger than 60 μm have fewer hydration products and may only have a filling effect in cement soil. The d90 of UFC particles in this test is less than 10 μm, mainly affecting cement soil strength at 28–90 d. Within this immersion time range, incorporating UFC enhances the chemical activity of cement particles, accelerating the hydration reaction speed. The degree of hydration is intense, generating more highly alkaline hydration products CSH (calcium silicate hydrate), CAH (Calcium aluminate hydrate), CASH (calcium aluminosilicate hydrate), Ca(OH)_2_. These alkaline substances neutralize the acidic environment produced by part or all of the fulvic acid impregnated in the internal pores of the sample so that the process of cement hydration reaction will not be affected in the short term. That is, the highly alkaline environment formed by the hydration of UFC and cement has a competing mechanism for filling the pores of cement soil by the rigid spherical colloid of fulvic acid. When the alkaline environment neutralizes the acidic environment of fulvic acid powerfully, the fulvic acid is impregnated inside the cement soil. The rigid spherical colloid of fulvic acid plays a leading role in cementing and filling the cement soil’s pores. The performance is as follows: with an increase in time, the strength of cement soil increases. When the neutralization of the acidic environment of fulvic acid by the alkaline environment is no longer so potent, the fulvic acid impregnated in the cement soil and the rigid spherical colloid of fulvic acid play a secondary role. The acidity of fulvic acid itself takes the dominant factor, which will weaken the hydration of cement and then reflect corrosion, manifested as the strength of cement soil gradually decreases with the increase of soaking time.

From the above analysis, it can be seen that the corresponding time of the peak strength of the sample in the fulvic acid soaking solution has a significant correlation with the hydration completion time of the composite curing agent and the time when the fulvic acid itself becomes corrosive. At 90 d, the UFC fine particles in the composite curing agent have completed the hydration, so the strength of the cement soil sample after 90 days is provided by the continuous hydration reaction of the aggregate particles formed by the ordinary cement in the composite curing agent and UFC fine particles. However, the change in cement particle size distribution and the increase in particle size will change the cement hydration rate and intensity. The amount of hydration products will also change under the same conditions, which causes a change in the alkaline environment. In this test, the fulvic acid reagent was continuously added to the saturated solution to control the constant pH value of the saturated solution. The insufficient neutralization ability of the alkaline environment inside the sample to the acidic environment of fulvic acid made the acidic environment of fulvic acid dominant, which highlighted the Corrosiveness, its adverse effect on cement hydration cannot be ignored, and the strength of cement soil will be reduced under long-term action.

However, for the cement soil sample soaked in distilled water with pH = 7.0, its strength continued to increase until it peaked when the soaking time reached 270 d. The strength of cement soil begins to decrease after soaking time exceeds 270 d. The reason is that in the strength development stage, the hydration reaction of cement continues and is not affected by the fulvic acid acidic soaking solution in the alkaline environment of cement hydration. Therefore, the hydration reaction makes the internal pore solution of the sample and the external immersion. The liquid environment is alkaline. The alkaline environment further promotes the reaction of cement particles and clay minerals with the alkaline solution, accelerates the hydration reaction, and the hydration products formed have an enhanced effect on the pore filling of the sample. The microscopic pore structure of the sample is dense, and the strength of the sample is improved [15]. As the immersion time increases, the alkaline environment of the immersion solution gradually increases.

Before the samples are taken from the immersion curing tank, the pH of the 270 d and 365 d water culture tanks is measured. It is found that the pH value in the soaking curing tank remains in the range of 11–12 inside. At this time, the pH value of the immersion water tank is relatively high, and the alkaline environment with a high pH value will affect the development of the strength of the cement soil. The strength of cement soil samples with a time of 365 days decreases. The research results of Yang Yuyou [16], Fan Gongjun [17], Mohsen Salehi [18], and Yan Li [19] also confirmed this conclusion. Secondly, humic acid has the characteristic of being slightly soluble in alkaline solution. The humic acid particles that constitute the soil skeleton will continuously dissolve in the highly alkaline environment generated by the hydration reaction. Especially when the immersion time reaches 270 days, the pH value of the immersion solution inside the curing box reaches 11–12, so the dissolution rate of humic acid particles is faster, resulting in a loose cement soil skeleton structure and a decrease in its strength value. However, the hydration of cement soil without humic acid reagent is not affected by humic acid, the alkaline environment inside the sample is robust, and the corrosion effect of the alkaline environment is also potent, so when the soaking time exceeds 270 d, its strength will also be reduced.

**Figure 10:**
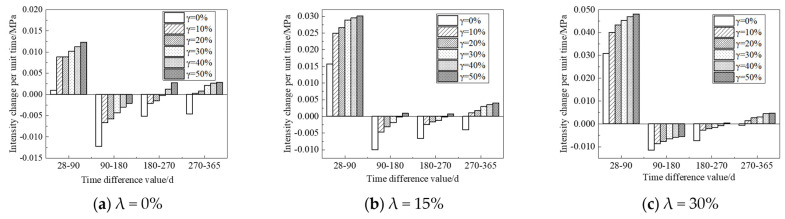
Histogram of the relationship between intensity change per unit time and soaking time (Fulvic acid solution pH = 5.0).

**Figure 11:**
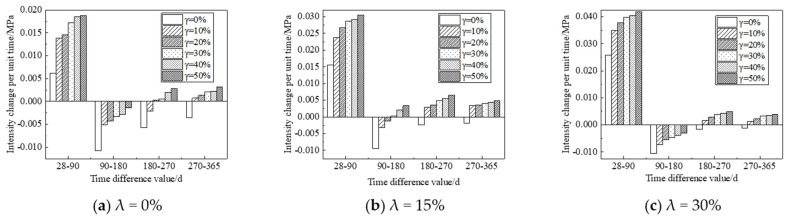
Histogram of the relationship between intensity change per unit time and soaking time (Fulvic acid solution pH = 6.0).

According to the figure above, UFC can significantly promote the enhancement of the strength of the sample in the strength enhancement stage. UFC can significantly inhibit the corrosion effect of fulvic acid on the strength of the sample in the corrosion stage of the sample. In order to accurately evaluate the influence of UFC content change on the corrosion resistance of cement soil samples in the fulvic acid soaking solution, the strength change per unit time is given for detailed analysis. It is used to evaluate the effect of UFC on the peat soil environment cement soil, the enhancement of the sample’s strength or the anti-corrosion effect’s size. When the strength variation per unit time is positive, the cement soil strength gradually increases within this period. Otherwise, the cement soil strength gradually decreases within this period. This study selects the sample’s strength change per unit time with a 20% cement mixing ratio for analysis, as shown in Figure 10 and Figure 11. The abscissa in the figure is the absolute value of the difference between the last and next soaking time, and the ordinate is the intensity change per unit of time. For example, the time difference (28–90) in Figure 10a corresponds to the intensity change per unit of time under different UFC dosages, which means that when the soaking time is 28 d and HA = 0%, the sample without UFC is taken as the initial strength, and the strength of the samples whose UFC content is 0%, 10%, 20%, 30%, 40%, and 50% when the immersion time is 90 d is, respectively, subtracted from the initial strength to obtain the strength difference. Then, the intensity change per unit of time is obtained by dividing the intensity difference by the absolute value of the time difference. In other cases, the calculation method of the intensity change per unit time is the same as above.

From Figure 10 and Figure 11, it can be seen that: (1) When the immersion time increases in the range of 28 d to 90 d, the strength of the sample shows an increasing trend, and they all reach the peak strength at 90 d; the most prominent contribution to the strength of the sample is when the UFC content is 10%. That is to say, UFC can enhance the strength of cement soil during the soaking period. (2) When the immersion time is more significant than 90 d, the strength of the samples without UFC shows a downward trend, indicating that the cement soil samples are corroded during this immersion period. The unit strength of the sample decreases gradually with the addition of UFC, and the unit strength decreases most significantly when the UFC content is 10% and the samples with a humic acid content of 0% and 15%. The unit strength reduction rate can reach more than 50%. With a further increase in UFC content, some samples’ unit strength reduction value becomes positive. That is, the strength value of the sample during this period exceeded the initial value, indicating that the addition of UFC greatly weakened the reduction of cement soil strength and significantly inhibited the corrosion of fulvic acid solution on cement soil samples, thus reflecting the excellent corrosion resistance of UFC.

**Figure 12:**
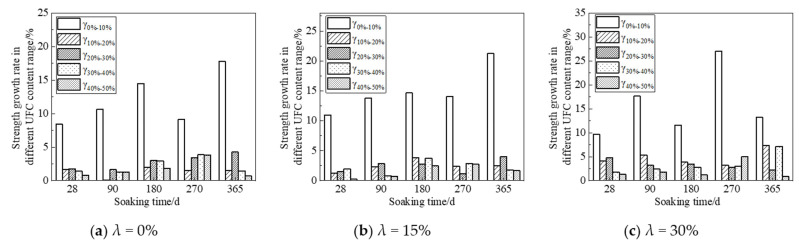
Relationship curve between strength growth rate and soaking time (Fulvic acid solution pH = 5.0).

**Figure 13:**
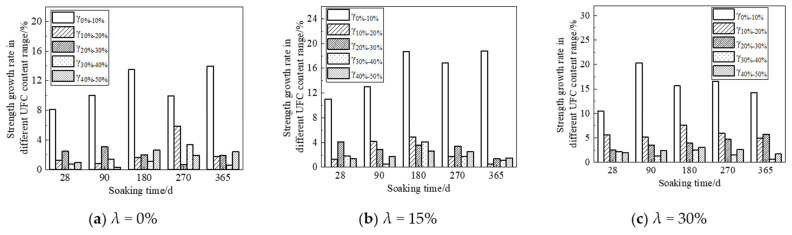
Relationship curve between strength growth rate of soaking time (Fulvic acid solution pH = 6.0).

The above analysis has demonstrated that adding UFC will significantly improve cement soil’s corrosion resistance in a peaty environment. In order to clarify the improvement effect of different UFC dosage ranges to find the best UFC dosage range, the strength data in Figure 7, Figure 8 and Figure 9 is converted into the robust growth rate within each UFC dosage range for analysis, and the calculation method is shown in Formulas (5) and (6). The calculated results are drawn into graphs, as shown in Figure 12 and Figure 13, where the abscissa is the soaking time, and the ordinate is the most robust growth rate of the samples in different UFC dosage ranges. Table 5 shows the strength reduction rate of samples with various UFC dosages within the range of 90 d to 365 d in the fulvic acid soaking solution with pH values of 5.0 and 6.0. The strength of the sample without UFC at 90 days is defined as the initial strength, and the initial strength of the samples with UFC content is 0%, 10%, 20%, 30%, 40%, and 50%, respectively, when the immersion time is 365 d. The difference is defined as the strength reduction value. Then the ratio of each strength reduction value to the initial strength is multiplied by 100% to obtain the strength reduction rate of the sample under each UFC content that changes with time.

(5) $$\Delta_{U}=U_{i+1}-U_{i}$$

(6) $$\delta_{U}=\frac{\Delta_{U}}{U_{i}}\times 100\%$$

In the formula: i = (0, 1, 2, 3, 4), Ui, Ui + 1 represents the strength of the sample when the UFC content is 0%, 10%, 20%, 30%, 40%, and 50%, respectively, MPa.

ΔU represents the difference between the strength corresponding to the latter dosage and the strength corresponding to the previous dosage within the range of UFC dosage of 0%, 10%, 20%, 30%, 40%, and 50%, MPa.

δU represents the most robust growth rate corresponding to different UFC dosage ranges, %. For example, UFC0%-10% indicates the corresponding strength growth rate when the UFC content increases from 0% to 10%.

From Figure 12 and Figure 13, it can be seen that at various soaking times and different humic acid contents, when the UFC content increases in the range of 0% to 10%, the most robust growth rate of the cement soil sample is far greater than other UFC content ranges. That is to say, UFC has the best effect on improving cement soil strength at this time. When the UFC content is greater than 10%, the most robust growth rate gradually decreases, and its effect on the strength of the sample is not apparent. In the environment of 365 days of soaking time, pH 5.0, and high fulvic acid content, the most robust growth rates of the samples in the range of 0~10% UFC content were 17.8%, 21.2%, and 13.2%, respectively. That is to say, in the long-term environment of high fulvic acid content, the addition of UFC can still bring about a minimum strength increase of 13.2%. It is enough to show that it has excellent long-term corrosion resistance to humic acid.

It can be concluded from Table 5 that in various humic acid environments, as the immersion time increases from 90 d to 365 d, the corrosion phenomenon of the sample without UFC is severe. The highest strength reduction rate can reach 54.7%. The value has exceeded half, which is unacceptable for engineering projects with strict requirements on durability. Under long-term action, the corrosion effect of humic acid on cement soil cannot be ignored, and corresponding measures must be taken to reduce the corrosion effect of cement soil. However, after adding 10% UFC, the strength reduction rate of the samples was significantly slowed down. The peat with high humic acid content was 20% cement, pH 6.0, and 30% humic acid reagent. Under the soil environment, the strength reduction rate of the sample decreased from 43.1% to 35.0% in 90–365 d. Compared with the same group of samples without UFC, the reduction rate of strength reduction can reach 18.8%. In the peat soil environment with a cement mixing ratio of 20%, pH value of 5.0, and high fulvic acid content without humic acid reagent, the strength reduction rate of the sample decreased from 43.6% to 33.6% at 90–365 d, compared with the same group of samples without UFC, the reduction rate of strength reduction can reach 22.9%. Even in the peat soil environment with the highest humic acid content at a pH value of 5.0 and a humic acid reagent addition of 30%, the strength reduction rate of the sample decreased from 54.7% to 48.7% at 90 days to 365 days, which was the same as that without. Compared with the same group of samples mixed with UFC, the reduction rate of strength reduction can reach 11.0%, but the reduction rate of strength reduction can be improved with the increase of UFC content. When the UFC content reaches 50%, the strength reduction rate of the sample at 90–365 d decreases from 54.7% to 39.2%. Compared with the same group of samples without UFC, the strength reduction rate can also reach 28.3%. In summary, no matter the case of high humic acid content, high fulvic acid content, or high humic acid content and fulvic acid content (high humic acid content), incorporating UFC can significantly improve the strength reduction rate under prolonged immersion time. With an increase in UFC content, the strength reduction rate is more significant, indicating that UFC plays an essential role in the long-term corrosion resistance of humic acid to cement soil and has excellent corrosion resistance.

The addition of UFC changes the particle size distribution of the composite curing agent, improves the chemical activity of cement particles, accelerates the hydration reaction speed, and improves the degree of hydration reaction. As the number of hydration products increases, the cementation between hydration products and soil particles increases, the pore diameter and connectivity of the internal pores of cement soil will be significantly reduced, and the overall structure will be more robust. The skeleton structure of the cement soil will become very dense, and the compactness of the microstructure will help the cement soil to resist the corrosion of various erosive media. Secondly, increased UFC content generates more highly alkaline hydration products in the solution, enhancing the alkaline environment. This alkaline environment will neutralize part of the inhibitory effect of humic acid on cement hydration and weaken the corrosive effect of humic acid on cement soil structure, thereby improving the strength and durability of the sample.

### 3.2. SEM and PCAS Test Results and Analysis

**Figure 14:**
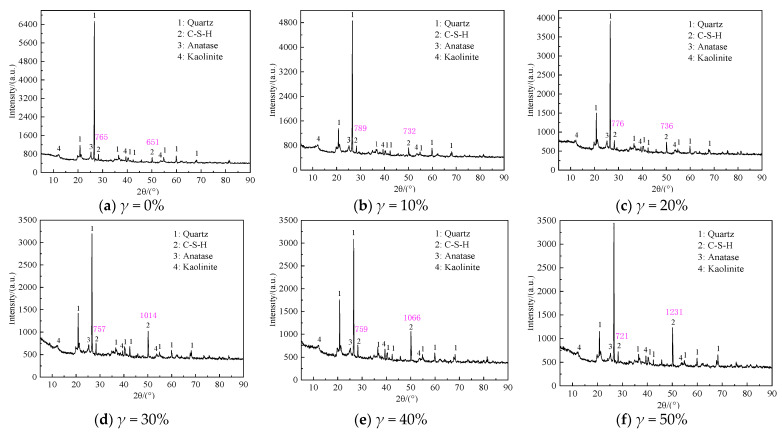
X-ray diffraction patterns of cement soil samples with different UFC content.

Figure 14a–f shows that when the mixing ratio of cement is 20%, the mixing amount of humic acid reagent is 15%. The pH = 6.0 is soaked in the fulvic acid solution for 90 days, with different UFC content (0%, 10%, 20%, 30%, 40%, 50%) X-ray diffraction patterns of samples.

Comparing the X-ray diffraction patterns of the cement soil samples with different UFC content in Figure 14a–f for phase analysis, it is found that no new characteristic peaks appear except for the known phases. With an increase in UFC content, the diffraction peaks of calcium silicate hydrate (2θ = 29.1°, 49.8°) gradually increase, and the relative content of hydration products increases slightly, which is consistent with the intensity results. Calcium silicate hydrate gel adheres to the surface of soil particles, cement. It entangles clay particles in the soil to form large aggregates and fill pores and plays a role in compacting the soil to a certain extent, which plays a role in developing the strength of cementitious materials. This result proves that UFC produces more hydration products than ordinary cement in a peaty soil environment, has a better curing effect than ordinary cement, and has better resistance to corrosive media.

### 3.3. SEM and PCAS Test Results and Analysis

**Figure 15:**
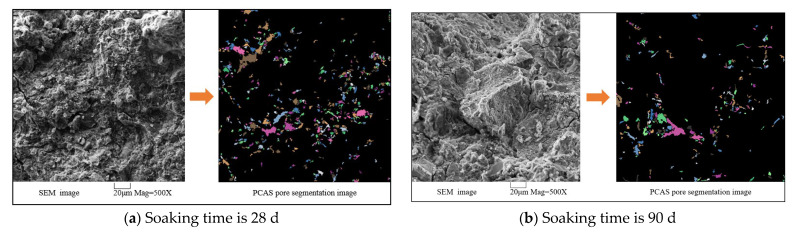

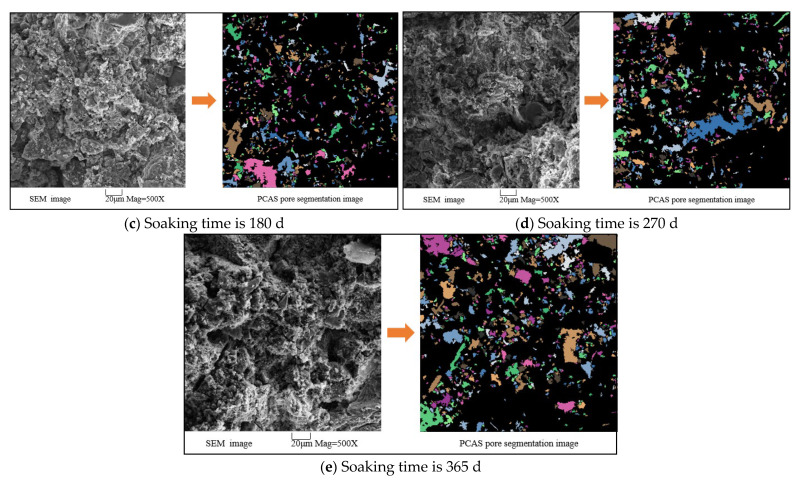
Microstructure images and PCAS pore segmentation processing images of cement soil under different immersion times.

In order to further explore the mechanism of UFC’s resistance to humic acid erosion under long-term immersion time, SEM tests and PCAS tests were carried out on cement soil samples with different immersion times. Figure 15 shows the 500-fold magnification of the cement soil samples with a humic acid content of 15%, soaking solution pH value of 6.0, UFC content of 10%, and soaking time of 28 d, 90 d, 180 d, 270 d, and 365 d, respectively. Typical SEM is processed by PCAS (Particles (Pores) and Cracks Analysis System) software. The operation steps of PCAS pore segmentation are as follows: firstly, the SEM image (500 times) is processed to filter out high-frequency noise and then imported into PCAS software for binary segmentation by setting parameters such as gray threshold, minimum pore area, and sealing radius. To automatically identify the pores and cracks in the image and obtain the pores’ geometric parameters and statistical parameters [20,21]. The pores are then segmented, while the particles are graded and identified with different colors. This test only conducts statistical analysis on large pores (particle size > 10 μm).

Figure 15a,b shows the microstructure images of the cement soil samples soaked for 28 d and 90 d. It can be seen that the interior of the sample is very dense, with solid structural connectivity and integrity. Dispersed granule units are less or almost invisible and tightly wrapped, filled, and cemented together by hydration products. Blocky hydration products overlap each other and accumulate layer by layer, filling the entire pores between large granules. Tiny pores are formed—an overall structural form with low connectivity. Figure 15c shows the microstructure images of the cement soil samples soaked for 180 d. It can be seen that the structural connectivity of the sample is slightly weakened. The cement soil structure has changed from a small pore low connectivity overall structure form to an aggregate-pore-aggregate connection form. The humic acid reaction consumes part of the hydration products between the aggregates. The filling and cementing effect is weakened to form pores in the overhead structure. However, there are no through pores with large pore diameters or many independent aggregate units, and the cement soil structure is still relatively dense. The pore size inside the cement soil increased slightly as the immersion time increased to 270 d and 365 d. However, the structural connectivity remained intact, as shown in Figure 15d,e. Part of the hydration products undergo a series of physical-chemical reactions with humic acid and then are consumed. The massive hydration products filled and cemented to form an overall structure are gradually eroded and transformed into a fibrous, rod-shaped network of hydration products. Its connection is weakened, the aggregate units are separated, and the pore structure gradually develops, forming a spatial network structure similar to a honeycomb. The figure has no sheets of large or interconnected pores; the overall structure remains with good connectivity.

The above results show that, for the cement soil samples that have been in the peat soil environment for a long time, due to the addition of UFC, its acceptable characteristics of small particle size, narrow particle size distribution, fast hydration reaction rate, high hydration degree and a large amount of hydration products can be brought into full play. Many excellent properties can be fully utilized, which gives the sample a higher early strength and a denser cement soil microstructure. Its ability to resist humic acid erosion is enhanced. With the increased immersion time, humic acid continued to erode into the sample’s interior. The excellent characteristics of UFC weakened the adverse effect of humic acid on the cement soil structure to a certain extent. Some hydration products were retained and continued to exert the filling and cementing effect to connect the aggregate units. The number of pores is significantly increased by erosion. However, the connectivity of the pores is not robust, and the integrity of the cement soil structure is still good. The above is evident because the excellent characteristics of UFC weaken the erosion effect of humic acid.

**Figure 16:**
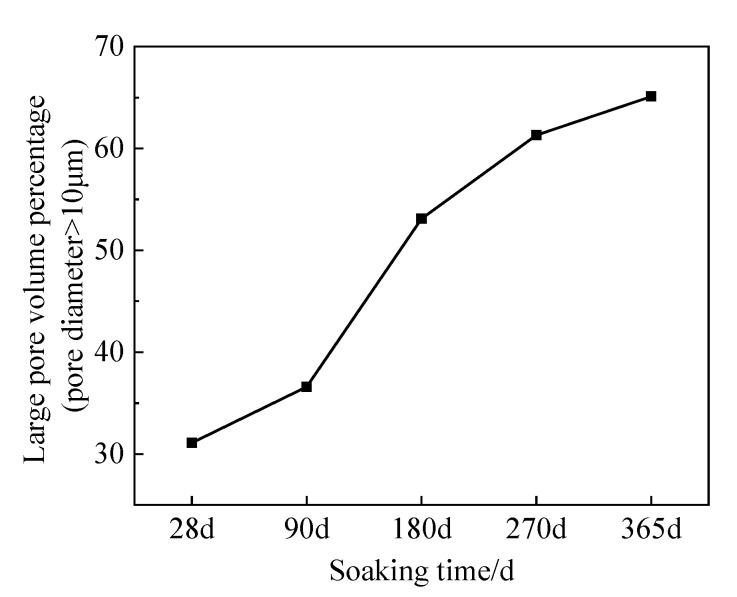
PCAS test results (>10 μm pore volume percentage).

The pore data parameters processed by PCAS software were summarized and counted to reveal the influence of UFC on the corrosion resistance of cement soil in the peat soil environment. Figure 16 shows the variation curve of the volume percentage of large pores (pore diameter > 10 μm) with the amount of UFC in the PCAS microscopic semi-quantitative test. It can be concluded from Figure 16 that with an increase in immersion time, the volume percentage of large pores shows an overall upward trend. The significant pore volume growth rate is the largest when the immersion time increases from 90 d to 180 d. When the immersion time exceeds 180 d, the growth rate of the volume percentage of large pores tends to be flat. Combined with the UCS test results, it can be seen that in the strength enhancement stage of 28–90 d, the hydration reaction of cement occupies the main factor, and the erosion effect of humic acid occupies the secondary factor, so the percentage of large pore volume increases slightly. When the immersion time exceeds 180 days, the erosion effect of humic acid is prominent, and the cement hydration reaction and filling and cementation of hydration products are weakened. Hence, the volume percentage of large pores increases significantly. Comprehensive SEM test and strength test (UCS) results found that an increase in the number of large pores did not significantly change the integrity of the cement soil structure. The cement soil can still resist humic acid erosion and has high strength.

## 4. Conclusions

This experiment mainly studies the effect of UFC content change on the corrosion resistance of cement soil in a peat soil environment under long-term immersion time. Adding different proportions of UFC to ordinary cement to form a composite curing agent aims to improve cement soil’s mechanical properties and durability through its excellent characteristics. Based on the strength test and microscopic test results, the mechanism of UFC’s anti-humic acid erosion was revealed, and the following conclusions were drawn:

(1) The UCS test results show that with the soaking time increase, the cement soil sample’s strength curve gradually increases to the peak strength and then decreases. That is, the strength change curve presents two stages: the strength-enhancing phase and the corrosion phase. The soaking times corresponding to the peak intensities were significantly different. With an increase in UFC content, the strength change per unit time of the sample, the growth rate of the strength of the unit UFC proportion, and the strength decrease in the rate of 90–360 d all show that compared with the sample without UFC, UFC can significantly reduce the intensity reduction rate of the sample. The sample can still maintain high strength under long-term immersion time, and the optimal dosage of UFC is 10%. It shows that UFC significantly inhibits the erosion of fulvic acid solution on cement soil samples, thus reflecting the excellent corrosion resistance of UFC.
(2) The results of SEM and PCAS tests show that the microstructure of cement soil gradually weakens with the increase in soaking time. However, the UFC particle size is small, the particle size distribution is narrow, the hydration reaction rate is fast, and the hydration degree is high. The excellent characteristics of high concentration and a large amount of hydration products weakened the adverse effects of humic acid on the cement soil structure to a certain extent. Although the number of large pores gradually increased, it did not significantly change the integrity of the cement soil structure. It still exhibits a relatively compact honeycomb overall structure, which correlates well with the strength test (UCS) results.
(3) The complete test results show that an increase in the UFC proportion will significantly improve cement soil’s durability in peat soil. Based on previous studies [3,4,5,6,7,8], this study adopts the test method of adding UFC to form a composite curing agent and discusses the influence of UFC content changes on the humic acid erosion res istance of cement soil in a peat soil environment from the perspective of strength test and microscopic test. Therefore, in future research and actual engineering of the peat soil environment in Dianchi Lake and Erhai Lake in Yunnan, we can combine the conclusions of this study with exploring more reasonable and effective ways to improve the mechanical properties and durability of cement soil in peat soil environment and reduce the amount of cement. Due to the significant CO_2_ emissions generated during cement production, the moderate use of UFC will also reduce total cement consumption and decrease CO_2_ emissions, promoting sustainable societal development.

## Figures and Tables

**Table 1:** Basic physical property indicators of test soil.

Test Soil	Natural Moisture Content (%)	Liquid Limit (%)	Plastic Limit (%)	Natural Density (g·cm^−3^)	The Specific Gravity of Soil Particles (Gs)
Cohesive Soil	18.60	39.20	23.00	1.96	2.84

**Table 2:** The chemical composition of the test soil and the mass fraction of each composition.

Test Soil	Chemical Composition and its Mass Fraction/%										
	SiO_2_	Fe_2_O_3_	Al_2_O_3_	TiO_2_	K_2_O	MgO	CaO	Na_2_O	MnO	P_2_O_5_	Loss on Ignition
Cohesive Soil	46.57	21.22	20.80	8.90	0.48	0.48	0.16	0.04	0.14	0.57	0.64

**Table 3:** Main chemical components of cement curing agent.

Material Type	Chemical Composition and Its Mass Fraction/%			
	CaO	SiO_2_	Al_2_O_3_	Fe_2_O_3_
OPC	65.5	18.4	5.3	2.9
UFC	65.5	18.0	5.4	2.9

**Table 4:** Test Plan.

Test Type	Cement Rate β/%	HA Content λ/%	Soaking Solution Type	Dosage of UFC γ/%	Soaking Time/d
UCS	20	0, 15, 30	FA solution (pH = 5.0, 6.0) Distilled water (pH = 7.0)	0, 10, 20, 30, 40, 50	28, 90, 180, 270, 365
SEM, PCAS	20	15	FA solution (pH = 6.0)	10	28, 90, 180, 270, 365

**Table 5:** In the fulvic acid soaking solution, the strength reduction rate of 90–365 d sample strength with the change of UFC content/%.

Cement Rate/β	Fulvic Acid Solution pH Value	HA. Content	90~365 d Sample Strength Reduction Rate/%					
			γ = 0%	γ = 10%	γ = 20%	γ = 30%	γ = 40%	γ = 50%
20%	pH = 5.0	*λ =* 0%	43.6	33.6	32.5	29.7	28.7	28.2
		*λ =* 15%	45.1	33.5	31.8	29.1	27.8	26.6
		*λ =* 30%	54.7	48.7	44.9	43.7	39.7	39.2
	pH = 6.0	*λ =* 0%	38.3	29.7	28.5	27.1	26.7	24.9
		*λ =* 15%	32.0	19.2	18.8	17.7	16.8	15.6
		*λ =* 30%	43.1	35.0	31.8	27.9	27.4	26.2

## Data Availability

The data used to support the finding of this study are included in the article.
